# Haptoglobin phenotype is not a predictor of recurrence free survival in high-risk primary breast cancer patients

**DOI:** 10.1186/1471-2407-8-389

**Published:** 2008-12-24

**Authors:** Marie-Christine W Gast, Harm van Tinteren, Marijke Bontenbal, René QGCM van Hoesel, Marianne A Nooij, Sjoerd Rodenhuis, Paul N Span, Vivianne CG Tjan-Heijnen, Elisabeth GE de Vries, Nathan Harris, Jos WR Twisk, Jan HM Schellens, Jos H Beijnen

**Affiliations:** 1 Department of Pharmacy & Pharmacology, the Netherlands Cancer Institute/Slotervaart Hospital, Amsterdam, the Netherlands; 2 Department of Biometrics, Antoni van Leeuwenhoek Hospital/the Netherlands Cancer Institute, Amsterdam, the Netherlands; 3 Department of Medical Oncology, Erasmus Medical Center – Daniel den Hoed Cancer Center, Rotterdam, the Netherlands; 4 Department of Medical Oncology, Radboud University Nijmegen Medical Center, Nijmegen, the Netherlands; 5 Department of Medical Oncology, University Medical Center Leiden, Leiden, the Netherlands; 6 Department of Medical Oncology, Antoni van Leeuwenhoek Hospital/the Netherlands Cancer Institute, Amsterdam, the Netherlands; 7 Department of Chemical Endocrinology, Radboud University Nijmegen Medical Center, Nijmegen, the Netherlands; 8 Division of Medical Oncology, Department of Internal Medicine, Maastricht University Medical Center, Maastricht, the Netherlands; 9 Department of Medical Oncology, University Medical Center Groningen, Groningen, the Netherlands; 10 Vermillion Inc, Fremont, CA, USA; 11 Department of Clinical Epidemiology and Biostatistics, VU University Medical Center, Amsterdam, the Netherlands; 12 Faculty of Science, Department of Pharmaceutical Sciences, Division of Biomedical Analysis, Utrecht University, Utrecht, the Netherlands

## Abstract

**Background:**

Better breast cancer prognostication may improve selection of patients for adjuvant therapy. We conducted a retrospective follow-up study in which we investigated sera of high-risk primary breast cancer patients, to search for proteins predictive of recurrence free survival.

**Methods:**

Two sample sets of high-risk primary breast cancer patients participating in a randomised national trial investigating the effectiveness of high-dose chemotherapy were analysed. Sera in set I (n = 63) were analysed by surface enhanced laser desorption ionisation time-of-flight mass spectrometry (SELDI-TOF MS) for biomarker finding. Initial results were validated by analysis of sample set II (n = 371), using one-dimensional gel-electrophoresis.

**Results:**

In sample set I, the expression of a peak at mass-to-charge ratio 9198 (relative intensity ≤ 20 or > 20), identified as haptoglobin (Hp) alpha-1 chain, was strongly associated with recurrence free survival (global Log-rank test; p = 0.0014). Haptoglobin is present in three distinct phenotypes (Hp 1-1, Hp 2-1, and Hp 2-2), of which only individuals with phenotype Hp 1-1 or Hp 2-1 express the haptoglobin alpha-1 chain. As the expression of the haptoglobin alpha-1 chain, determined by SELDI-TOF MS, corresponds to the phenotype, initial results were validated by haptoglobin phenotyping of the independent sample set II by native one-dimensional gel-electrophoresis. With the Hp 1-1 phenotype as the reference category, the univariate hazard ratio for recurrence was 0.87 (95% CI: 0.56 – 1.34, p = 0.5221) and 1.03 (95% CI: 0.65 – 1.64, p = 0.8966) for the Hp 2-1 and Hp 2-2 phenotypes, respectively, in sample set II.

**Conclusion:**

In contrast to our initial results, the haptoglobin phenotype was not identified as a predictor of recurrence free survival in high-risk primary breast cancer in our validation set. Our initial observation in the discovery set was probably the result of a type I error (*i.e*. false positive). This study illustrates the importance of validation in obtaining the true clinical applicability of a potential biomarker.

## Background

Following lung cancer, breast cancer currently is the second leading cause of cancer deaths in women [1]. A substantial survival benefit is achieved by treatment with adjuvant systemic therapy. The main prognostic factors in breast cancer include clinical (age) and pathological parameters (tumour size, lymph node status, and grade of malignancy), whereas the hormone-receptor and Her2/neu-receptor status are (also) predictive factors [2]. However, 30 – 50% of high-risk primary breast cancer patients will eventually develop metastatic relapse and die, despite locoregional treatment and adjuvant systemic chemotherapy, while there is a small percentage that would have survived without adjuvant chemotherapy and hormonal therapy [3]. Clearly, improved breast cancer prognostication is urgently needed to more accurately predict clinical outcome in individual patients and as such reduce both over- and undertreatment of the disease.

High-throughput genomic and transcriptomic approaches have recently demonstrated to generate signatures that better predict clinical outcome than conventional prognosis criteria. For example, investigators from our respective institutes have published gene expression profiles in tumour tissue that outperformed all clinical variables in predicting disease outcome (risk of recurrence) [4-7]. Similarly, a RT-PCR based multigene assay was recently shown to accurately predict both the probability of recurrence and the magnitude of chemotherapy benefit in node-negative, oestrogen-receptor positive breast cancer [8].

An alternative and complementary approach is to perform protein expression analysis. As the proteome reflects gene expression as well as protein stability and post-translational modifications, protein data could, in principle, be used for the same purpose. One of the techniques currently applied in proteomics research of breast cancer is surface-enhanced laser desorption ionisation time of flight mass spectrometry (SELDI-TOF MS). Until now, only two studies have been published in which this platform was applied in the identification of serum markers for prognosis of breast cancer [9,10]. Comparing the tumour cytosolic extract of node-negative sporadic breast tumours with or without a recurrence, Ricolleau et al. [10] identified a high level of ubiquitin and/or a low level of ferritin light chain to be associated with a good prognosis in breast cancer (n = 60). Goncalves et al. [9] constructed a multiprotein model, consisting of 40 proteins, that correctly predicted relapse in 67 of the 81 patients of which fractionated sera were investigated. These promising results need to be interpreted cautiously, as in both studies only a limited number of patients was investigated, and results have not been validated yet by analysis of independent study populations.

Hence, the aim of the current study is to investigate sera of high-risk primary breast cancer patients to search for proteins predictive of recurrence free survival, and to validate our results by analysis of an independent study population.

## Methods

### Study population

From 1993 to 1999, high-risk primary breast cancer patients who had undergone modified radical mastectomy or breast conserving surgery with complete axillary clearance participated in a randomised multicentre phase III trial. This study investigated the benefit of high-dose adjuvant chemotherapy in patients with ≥ 4 axillary lymph node metastases. The design of the study has been described elsewhere [11]. Major eligibility criteria were histologically confirmed stage IIA, IIB or IIIA breast cancer with at least 4 tumour-positive axillary lymph nodes but no evidence of distant metastases, age under 56 years, and no previous other malignancies.

In sample set I, sera of 63 study patients who were treated in the Netherlands Cancer Institute were included. Sera were procured after surgery (7 – 51 days), but prior to adjuvant chemotherapy (0 – 45 days). All sera were obtained and stored under strictly defined conditions at the Institutional Serum Bank. In sample set II, serum/plasma samples (procured at any time point in therapy) of 371 study patients treated in the Netherlands Cancer Institute (sera; n = 15, plasma; n = 38), the Erasmus Medical Center – Daniel den Hoed Cancer Center (sera; n = 114), the Radboud University Medical Center Nijmegen (sera; n = 87), the University Medical Center Groningen (sera; n = 69), and the University Medical Center Leiden (sera; n = 48) were included. All samples were obtained with medical-ethics approval and all patients gave informed consent.

### Chemicals

All used chemicals were obtained from Sigma, St. Louis, MO, USA, unless stated otherwise.

### Biomarker discovery

Protein profiling was performed using the ProteinChip SELDI Reader (Bio-Rad Laboratories, Hercules, CA, USA). Several chromatographic array surfaces with suitable binding conditions were screened for discriminative mass-to-charge ratio's (m/z) between unfractionated sera of breast cancer patients of set I either experiencing a recurrence at a relatively short follow-up (Recurrence Free Survival (RFS) < 16 months, n = 4), or experiencing no recurrence after a long follow-up (> 75 months, n = 4). Optimal discrimination between both groups was obtained by Q10 arrays (strong anion exchange chromatography) with 100 mM Tris-HCl pH 8/0.1% TritonX-100 as a binding buffer. This assay was subsequently applied in the analysis of all sera in sample set I (n = 63).

In brief, samples were thawed on ice and denatured by 1:10 dilution in 9 M urea/2% 3- [(3-cholamidopropyl)dimethylammonio]-1-propanesulfonate (CHAPS)/1% dithiotreitol (DTT). Arrays were assembled in a 96-well bioprocessor (Bio-Rad Labs), which was placed on a platform shaker at 350 rpm at all steps of the protocol. Arrays were equilibrated twice with 200 μl of binding buffer for 5 min. Pretreated serum samples were diluted 1:10 in binding buffer and were randomly applied to the arrays. After a 30 min incubation, the arrays were washed twice with binding buffer and twice with 100 mM Tris-HCl pH 8 for 5 min. Following a quick rinse with deionised water (Braun, Emmenbrücke, Germany), arrays were air-dried. A 50% solution of sinapinic acid (Bio-Rad Labs) in 50% acetonitrile (ACN)/0.5% trifluoroacetic acid (TFA) was applied twice (1.0 μl) to the array as matrix. Following air-drying, the arrays were analysed using the ProteinChip SELDI (PBS IIc) Reader (Bio-Rad Labs). For mass accuracy, the instrument was calibrated on the day of measurements with All-In-One peptide standard (Bio-Rad Labs). Data were collected between 0 and 200 kDa, averaging 65 laser shots with intensity 158, detector sensitivity 5, and a focus lag time of 746 ns. Spectra were baseline subtracted and normalised to the total ion current from 1.5 to 200 kDa. The Biomarker Wizard software package (version 3.1, Bio-Rad Labs) was applied for peak detection. Peaks were auto-detected when occurring in at least 25% of spectra and when having a signal-to-noise ratio of at least 5. Peak clusters were completed with peaks with a signal-to-noise ratio of at least 2 in a cluster mass window of 0.3%.

### Biomarker characterisation

A 500 μl serum sample containing the biomarker of interest marker (*i.e*. m/z 9198) was denatured in 9 M urea/2% CHAPS/1% DTT in 50 mM Tris-HCl pH 9. The sample was subsequently fractionated on Q Ceramic HyperD beads with a strong anion exchange moiety (Biosepra Inc., Malborough, MA, USA). After binding of denatured sample to the beads, the flow through was collected and bound proteins were subsequently eluted with buffers of pH 9 – 3. The fraction containing the marker was further purified by size fractionation, using Microcon 50 kDa MW spin concentrators (YM50, Millipore, Billerica, MA, USA) with increasing concentrations of ACN/0.1% TFA. The filtrate containing the m/z 9198 marker was subsequently de-salted by application on reversed phase RP18 beads (Varian Inc., Palo Alto, CA, USA), followed by elution with increasing concentrations of ACN containing 0.1% TFA. The purification process was monitored by profiling each fraction on Q10 arrays and NP20 arrays (a non-selective, silica chromatographic surface). Eluates containing the m/z 9198 marker were dried and redissolved in loading buffer for SDS-PAGE, which was performed on Novex NuPage gels (18% Tris-Glycine gel; Invitrogen, San Diego, CA, USA). Following Coomassie staining (Simply Blue; Invitrogen), protein bands of interest were excised and collected. The proteins within the excised bands were eluted by washing twice with 30% ACN/100 mM ammonium bicarbonate, followed by dehydration in 100% ACN. Gel bands were subsequently heated at 50°C for 5 min and eluted with 45% formic acid/30% ACN/10% isopropanol under sonification for 30 min. After leaving the eluates overnight at room temperature, they were profiled on NP20 arrays. Eluates were subsequently dried, resuspended in 20 ng/μl trypsin (Promega, Madison, WI, USA) in 10% ACN/25 mM ammonium bicarbonate, followed by incubation at room temperature for 4 h for protein digestion. For in-gel protein digestion, gel bands were first washed with 40% methanol/10% acetic acid twice, followed by a 30% ACN/100 mM ammonium bicarbonate wash. Gel bands were dried by SpeedVac and digested for 12 h by trypsin (20 ng/μl 100 mM ammonium bicarbonate). All tryptic digests were profiled on NP20 chips, using 1 μl 20% alpha-cyano-4-hydroxy cinnaminic acid solution in 50% ACN/0.5% TFA as matrix. Peptides in the digests were investigated with the NCBI database using the ProFound search engine at  with the following search parameters: standard cleavage rules for trypsin, 1 missed cleavage allowed. Confirmation of protein identity was provided by sequencing tryptic digest peptides by quadrupole-TOF (Q-TOF) MS (Applied Biosystems/MSD Sciex, Foster City, CA, USA) fitted with a ProteinChip Interface. Fragment ion spectra resulting from Q-TOF analyses were taken to search the SwissProt 44.2 database (Homo Sapiens: 11072 sequences) using the MASCOT search engine at  (Matrix Science Ltd., London, UK), with the following search parameters: monoisotopic precursor mass tolerance: 40 ppm, fragment mass tolerance: 0.2 Da, variable modifications: methionine oxidation, and trypsin cleavage site. Throughout the identification experiments, a serum sample lacking the m/z 9198 marker was run concurrently as a negative control.

### Haptoglobin phenotyping assay

**Figure 1:**
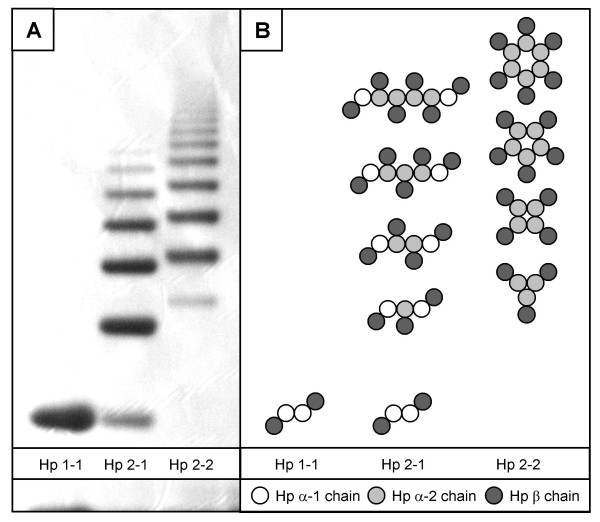
**Haptoglobin phenotype assessment using a native one-dimensional gel electrophoresis system**. A: Specific migration pattern of Hp 1-1, Hp 2-1, and Hp 2-2 in a 3 – 8% gradient Tris-Acetate gel. B: Composition of the three haptoglobin phenotypes Hp 1-1, Hp 2-1, and Hp 2-2 (adapted from [14]).

The haptoglobin (Hp) phenotype of all samples in set I and II was assessed by native one-dimensional gel electrophoresis, followed by peroxidase staining. One μl of serum or plasma sample was mixed with 19 μl of a 1:100 dilution of haemolysate in phosphate buffered saline. Following incubation for 5 min at room temperature, 10 μl of 3× native sample buffer (30 ml glycerol/18.8 ml 1 M Tris-HCl pH 6.8/1.5 ml 1% (w/v) bromophenol blue, made to 100 ml with water) was added and mixed. Samples were then loaded onto a 3 – 8% gradient Tris-Acetate NuPAGE precast gel (Invitrogen, Karlsruhe, Germany). Samples were run at a constant 150 V, gradient 18 – 7 mA for 3 h, using a running buffer of 25 mM Tris/250 mM glycine, adjusted to pH 8.6. After staining with 1% (w/v) rhodamine 1%, the gel was incubated for 10 min in a 1:1 water-diluted leucomalachite green peroxidase-development buffer (0.2 g leucomalachite green/0.02 g EDTA in 25 ml 40% (v/v) acetic acid with 0.06% (v/v) H_2_O_2_). The phenotype of each sample was subsequently determined by its specific migration pattern, which appears as black bands in the gel (Figure 1A) [12].

### Statistical analysis

Survival curves were analysed according to the Kaplan-Meier method from the date of randomisation to the time of first recurrence or death, or the date of last follow-up. The curves were compared by log-rank statistics. To investigate the relation of haptoglobin phenotype and other variables with recurrence-free survival time, a Cox proportional hazards model was used. Relations were expressed in terms of hazard ratios with 95% confidence intervals. Possible confounding clinical variables that either have known prognostic or predictive value (*i.e*. treatment (high dose vs. conventional dose chemotherapy), age (≥ 40 yrs vs. < 40 yrs), number of positive lymph node (0 – 9 vs. ≥ 10), tumour size (< 5 cm vs. ≥ 5 cm), Her2/Neu status (negative vs. positive, of note, patients did not yet receive adjuvant trastuzumab), receptor status (oestrogen and/or progesterone receptor (ER/PR) positive vs. negative), and Bloom-Richardson grade (grade I vs. grade II vs. grade III)), or variables that were related to the exposure haptoglobin phenotype (*i.e*. surgery (breast conserving vs. mastectomy)) were incorporated into the model.

The distribution of patient characteristics over the two sample sets were compared using either the Chi-square test or the Fisher's exact test for categorical variables and the Mann-Whitney U test for continuous variables. All statistical analyses were performed using SPSS statistical software, version 13.0 (SPSS Inc., Chicago, IL, USA) and SAS statistical software, version 9.1.3 (SAS Institute Inc., Cary, NC, USA). Statistical tests were two sided at the 5% level of significance.

## Results

### Study population

At time of analysis, in sample set I (n = 63), 28 patients had a recurrence or had died and 35 patients were censored at a median follow-up of 6.6 years. In sample set II (n = 371), 149 patients had a recurrence or had died and 222 patients were censored at a median follow-up of 8.0 years. Characteristics of both sample sets are provided in Table 1. All patient characteristics were similarly distributed between sample set I and sample set II, as determined by the Chi-square test or the Mann-Whitney U test.

**Table 1:** Patient and tumour characteristics of sample set I and II

	**Sample set I **(n = 63)		**Sample set II **(n = 371)	
**Patient characteristics**	N	(%)	N	(%)

Age				

Mean [range]	45.8	[33–55]	43.9	[26–55]

< 40 years	10	(16%)	94	(25%)

≥ 40 years	53	(84%)	277	(75%)

Menopausal status				

Premenopausal	49	(78%)	317	(85%)

Postmenopausal	11	(17%)	40	(11%)

Unknown	3	(5%)	14	(4%)

Surgery				

Mastectomy	56	(89%)	291	(78%)

Breast conserving	7	(11%)	80	(22%)

Treatment				

Conventional dose	27	(43%)	158	(43%)

High dose	36	(57%)	213	(57%)

				

**Tumour characteristics**				

Number of positive lymph nodes				

4 – 9	40	(63%)	241	(65%)

≥ 10	23	(37%)	130	(35%)

Tumour size				

T1 (< 2 cm)	9	(14%)	90	(24%)

T2 (2 – 5 cm)	41	(65%)	225	(61%)

T3 (= 5 cm)	13	(21%)	56	(15%)

Her2/Neu status				

Negative	42	(67%)	274	(74%)

Positive	16	(25%)	81	(22%)

Unknown	5	(8%)	16	(4%)

Oestrogen/Progesterone receptor status				

ER and PR negative	10	(16%)	101	(27%)

ER and/or PR positive	50	(79%)	250	(68%)

Unknown	3	(5%)	20	(5%)

Bloom-Richardson grade				

Grade I	13	(21%)	62	(17%)

Grade II	26	(41%)	112	(30%)

Grade III	20	(32%)	170	(46%)

Unknown	4	(6%)	27	(7%)

### Biomarker discovery

**Figure 2:**
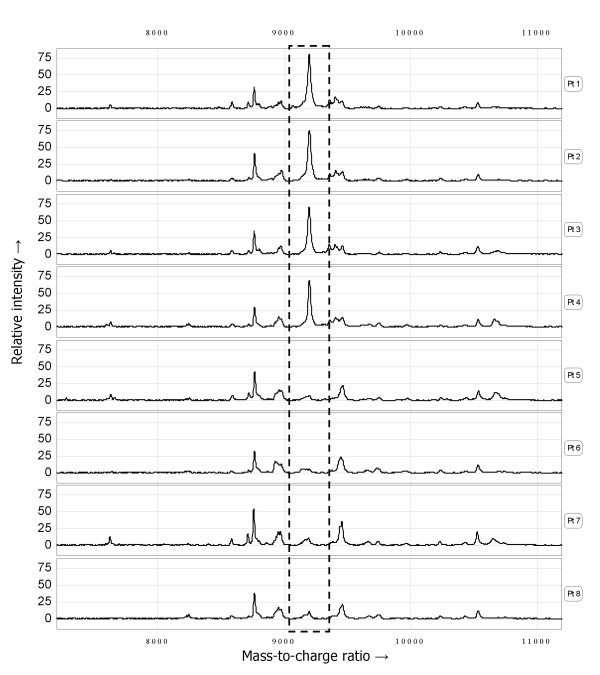
**Representative example of serum protein profiles (sample set I) obtained with the optimized SELDI-TOF MS assay, showing the clear dichotomous expression of the m/z 9198 peak (dotted box)**.

**Figure 3:**
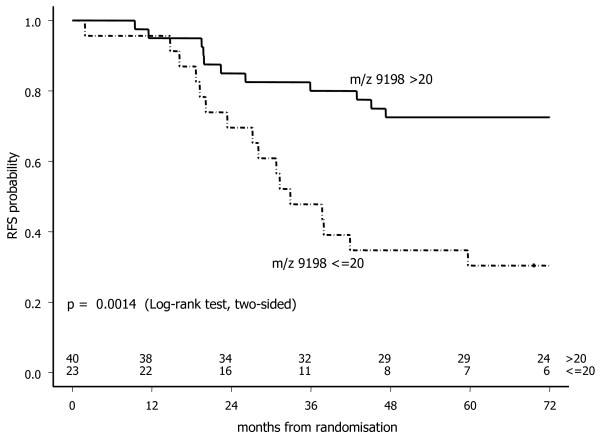
**Recurrence free survival in sample set I (n = 63) according the m/z 9198 peak intensity > 20 or ≤ 20, as determined by SELDI-TOF MS**.

Following evaluation of several chromatographic array surfaces with suitable binding conditions, the Q10 array with 100 mM Tris-HCl pH 8/0.1% TritonX-100 as a binding buffer gave optimal results in our screening population (n = 8). Using this SELDI-TOF MS assay, the spectra of sera from patients experiencing no recurrence (n = 4) could clearly be distinguished by the spectra of sera from patients that recurred at a relatively short follow-up (n = 4) by overexpression of a peak at m/z 9198. The clear dichotomous distribution in the relative m/z 9198 peak intensity was subsequently confirmed in the acquired mass spectra of all 63 sera in sample set I (peak intensity > 20: n = 40, ≤ 20: n = 23). Representative SELDI-TOF MS spectra are presented in Figure 2. The Kaplan-Meier curve (Figure 3) shows a significant difference in the probability of remaining recurrence free (Log-rank test, p = 0.0014) between high-risk primary breast cancer patients exhibiting a peak at m/z 9198 with a relative intensity > or ≤ than 20. The univariate hazard ratio was 3.22 (95% CI: 1.51 – 6.85, p = 0.0024).

### Biomarker characterisation

**Figure 4:**
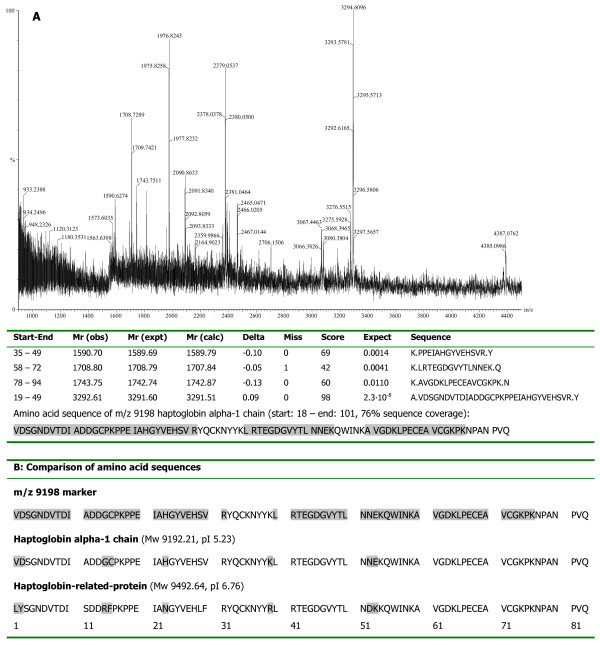
**Structural identification of the m/z 9198 peak cluster**. A. Peptide mapping of the m/z 9198 marker. MS spectrum of the m/z 9198 tryptic digest in the gel eluate. All peptides were sequenced with tandem MS using Q-TOF for confirmation. Results from the MASCOT search for protein identification include start and end positions of the peptide sequence starting from the amino acid terminal of the whole protein, the observed m/z, transformed to its experimental mass (Mr(expt)), the calculated mass (Mr(calc)) from the matched peptide sequence, as well as their mass difference (Delta), the number of missed cleavage sites for trypsin (Miss), the estimate Z-score and its significance, and the peptide sequence. B. Matched amino acid sequence of the m/z 9198 marker (in grey: amino acid sequence sequenced by Q-TOF MS), haptoglobin alpha-1 chain and the corresponding N-terminus of haptoglobin-related-protein (in grey: amino acid substitutions between haptoglobin and haptoglobin-related-protein) [24].

Following anion exchange fractionation, the m/z 9198 marker was eluted in the pH 5 eluate. This fraction was concentrated on YM50 spin concentrators, and the marker was found in the water wash. De-salting of the water wash on RP18 beads resulted in elution of the marker in the 60% ACN/0.1% TFA eluate, which was subsequently subjected to SDS-PAGE analysis. After staining, a clear band in the 9 kDa region was visible, which was excised and subjected to passive elution followed by tryptic digestion of the eluate. Profiling of the gel-eluate confirmed the presence of the marker. Peptide mapping of the tryptic digest identified the marker as haptoglobin alpha-1 chain (estimated Z-score 1.49, 48% sequence coverage), which is an 83 amino acid peptide with a theoretical mass of 9192.21 Da and a pI of 5.23. This identity [Swiss-Prot:P00738] was confirmed by amino acid sequencing of 4 peptides in the tryptic digest by tandem MS on a Q-TOF (76% coverage, Figure 4A).

**Figure 5:**
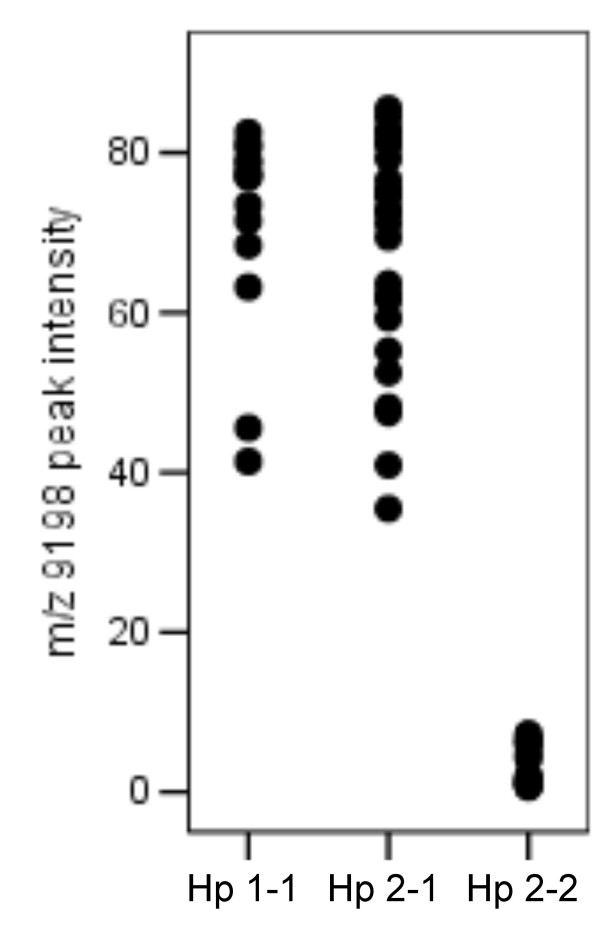
**Peak intensity of the m/z 9198 marker (as determined by SELDI-TOF MS) vs. haptoglobin phenotype (as assessed by 1D gel-electrophoresis) of samples in set I**.

Haptoglobin occurs *in vivo *as polymers of an alpha and beta chain complex, interlinked via disulfide bridges. There are two major alpha chains: alpha-1 (83 amino acids, 9.2 kDa) and alpha-2 (142 amino acids, 16 kDa), of which the alpha-2 chain is the product of unequal crossing over between two alpha-1 alleles [13]. Due to this genetic variation, haptoglobin occurs in three major (pheno)types: Hp 1-1, Hp 2-1 and Hp 2-2, occurring in 16%, 48%, and 36%, respectively, of the northwestern European population [14]. The Hp 1-1 phenotype consists of an [alpha-1 – beta] dimer (86 kDa), whereas Hp 2-1 consist of two [alpha-1 – beta] units flanking a variable length [alpha-2 – beta] polymer (86 – 300 kDa). Hp 2-2, the largest species, consists of multiple repeats of an [alpha-2 – beta] unit (170 – 900 kDa) (Figure 1B) [15,16]. Expression of the Hp alpha-1 chain, as determined by SELDI-TOF MS, will correspond to the actual haptoglobin phenotype, since the haptoglobin alpha-1 chain is only expressed by individuals with the Hp 1-1 or Hp 2-1 phenotype [12]. Indeed, following haptoglobin phenotype assessment by native one-dimensional gel-electrophoresis, all patients with m/z 9198 = 20 carried the Hp 2-2 phenotype (n = 23), while patients with m/z 9198 > 20 were shown to have either the Hp 1-1 (n = 14) or Hp 2-1 (n = 26) phenotype (Figure 5).

**Figure 6:**
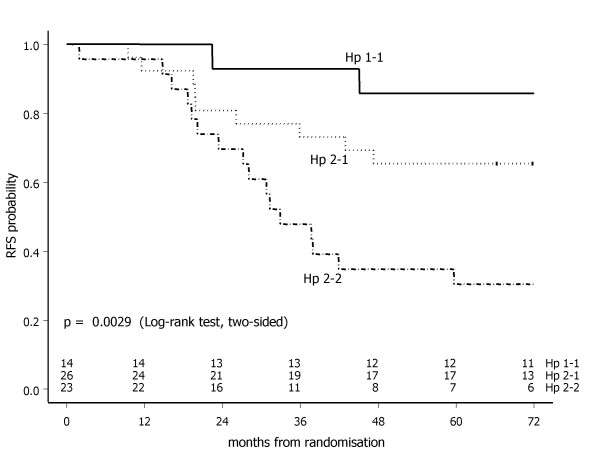
**Recurrence free survival in sample set I (n = 63) by haptoglobin phenotype**.

Following Kaplan-Meier analysis by haptoglobin phenotype in sample set I (n = 63), the Hp 1-1, 2-1 and 2-2 phenotypes were shown to be associated with a good, intermediate and poor prognosis, respectively (global Log-rank test, p = 0.0029) (Figure 6). With Hp 1-1 phenotype as the reference category, the univariate hazard ratio was 3.08 (95% CI: 0.67 – 14.10, p = 0.1464) for Hp 2-1, and 7.37 (95% CI: 1.69 – 32.23, p = 0.0079) for Hp 2-2 phenotype. In the multivariate Cox regression analysis, haptoglobin phenotype was independently associated with recurrence free survival (Hp 2-2; p = 0.0098), while for receptor status (ER/PR negative; p = 0.0962) and treatment arm (conventional dose; p = 0.0509) a borderline significant association was observed (Table 2).

**Table 2:** Multivariable proportional-hazards analyses for the risk of recurrence for patients in sample set I and II

**Variable**	**Sample set I **(n = 63)			**Sample set II **(n = 371)		
	
	HR^a^	(95% CI^b^)	p-value	HR^a^	(95% CI^b^)	p-value
Haptoglobin phenotype						

Hp 1-1	1	-	-	1	-	-

Hp 2-1	4.21	(0.44 – 39.83)	0.2105	0.94	(0.58 – 1.52)	0.8059

Hp 2-2	17.76	(2.00 – 157.44)	0.0098	1.26	(0.76 – 2.08)	0.3653

Surgery						

Breast conserving	1	-	-	1	-	-

Mastectomy	1.26	(0.24 – 6.67)	0.7843	0.91	(0.59 – 1.39)	0.6514

Treatment arm						

High dose	1	-	-	1	-	-

Conventional dose	0.33	(0.11 – 1.00)	0.0509	1.36	(0.96 – 1.92)	0.0809

Age						

≥ 40 yrs	1	-	-	1	-	-

< 40 yrs	1.26	(0.32 – 4.93)	0.7413	0.97	(0.65 – 1.46)	0.8890

No. of positive lymph nodes						

4 – 9	1	-	-	1	-	-

≥ 10	0.72	(0.23 – 2.29)	0.5785	1.00	(0.69 – 1.45)	0.9964

Tumour size						

< 5 cm	1	-	-	1	-	-

≥ 5 cm	1.35	(0.50 – 3.65)	0.5497	1.63	(1.03 – 2.60)	0.0374

Her2/Neu status						

Negative	1	-	-	1	-	-

Positive	1.47	(0.53 – 4.08)	0.4641	1.47	(0.99 – 2.20)	0.0589

Receptor status						

ER/PR positive	1	-	-	1	-	-

ER/PR negative	3.39	(0.80 – 14.27)	0.0962	1.11	(0.73 – 1.70)	0.6138

Bloom-Richardson grade						

Grade I	1	-	-	1	-	-

Grade II	0.92	(0.26 – 3.23)	0.9016	0.87	(0.50 – 1.50)	0.6187

Grade III	1.28	(0.37 – 4.46)	0.6946	1.34	(0.79 – 2.26)	0.2796

^a^HR: Hazard Ratio, ^b^CI: Confidence Interval

### Biomarker validation

The distribution of the haptoglobin phenotype of patients in the validation sample set II (n = 371) was subsequently assessed for validation purposes. As the haptoglobin phenotype (i.e. genotype) is not influenced by treatment, samples in set II were collected at any time point in therapy. All patient characteristics were similarly distributed between sample set I and sample set II. The Hp 1-1, 2-1, and 2-2 phenotype was determined in 70, 189, and 112 patients, respectively, yielding an allele frequency of 0.44, which is in concordance with previously reported frequencies. [14]

**Figure 7:**
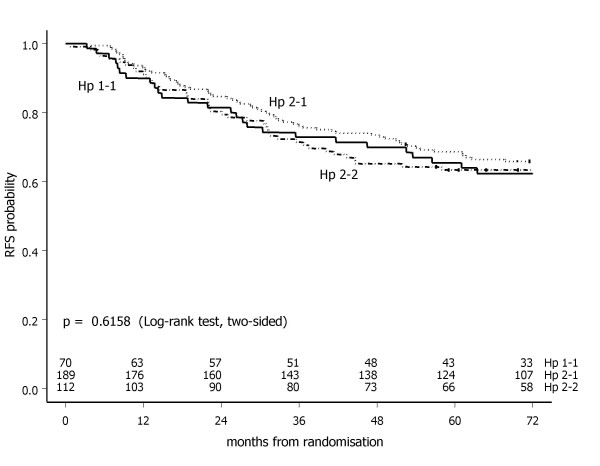
**Recurrence free survival in sample set II (n = 371) by haptoglobin phenotype**.

The Kaplan-Meier curve, however, did not show a significant difference in the probability of recurrence free survival (global Log-rank test, p = 0.6158) between the high-risk primary breast cancer patients in sample set II having the Hp 1-1, 2-1 or 2-2 phenotype (Figure 7). With the Hp 1-1 phenotype as the reference category, the univariate hazard ratio was 0.87 (95% CI: 0.56 – 1.34, p = 0.5221) and 1.03 (95% CI: 0.65 – 1.64, p = 0.8966) for the Hp 2-1 and Hp 2-2 phenotypes, respectively. This finding was not affected by tumour size, which was found to be the only independently associated variable for recurrence free survival (p = 0.0374). Her2/Neu status (p = 0.0589) and treatment arm (p = 0.0809) were only borderline significantly associated with recurrence free survival in set II (Table 2).

## Discussion

The introduction of high-throughput analytical platforms, such as the genomic/transcriptomic microarray technology, or the proteomic SELDI-TOF MS technology, has enabled the advent of discovery-based research. Large quantities of data can now be analysed without underlying hypotheses, to search for patterns that discriminate between patients with different diagnosis, prognosis or response to treatment. Assessment of validity, however, is pivotal in this discovery-based '-omics' research, as the meaning of such patterns from a biological perspective often is unknown.

Our initial findings were, however, endorsed by the various biological functions of haptoglobin and their phenotype-dependency. The main physiological function of haptoglobin is binding of free haemoglobin. The haptoglobin-haemoglobin complex is too large to be filtered at the kidney glomerulus and is therefore retained. Both iron loss and free-radical mediated damage, caused by the haem-iron mediated generation of free hydroxyl radicals (by means of the Fenton reaction: H_2_O_2 _+ Fe^2+ ^→ Fe^3+ ^+ OH^- ^+ ^·^OH) are thus prevented [14]. Haptoglobin has also been identified as a strong angiogenic agent, activating endothelial cell growth and differentiation. This function was shown to be phenotype dependent, as the Hp 2-2 phenotype has been found to be more angiogenic than the other phenotypes [17]. The poor prognosis of our Hp 2-2 breast cancer patients in our discovery set (n = 63) could be exerted via this haptoglobin function, since angiogenesis is well known to be involved tumor growth, proliferation, and metastasis [18].

Both haptoglobin and its phenotype have been described previously in relation to various diseases (including cancer), a finding which is not surprising in view of its biology. Both the intact protein and its subunits have been found overexpressed in serum of patients with various solid tumours, for example ovarian and small-cell lung cancer [19-21]. The 9.2 kDa haptoglobin alpha-1 chain has been specifically detected by Tolson et al. [12] in sera of renal cell carcinoma patients and healthy controls. Following haptoglobin phenotyping, all patients having the Hp 2-2 phenotype indeed proved to be missing the 9.2 kDa haptoglobin alpha-1 peak in their serum protein profile. Due to its phenotypic distribution, this protein could, however, not be considered as a diagnostic marker. The influence of haptoglobin phenotype on recurrence free survival has not been investigated. [12] Using the SELDI-TOF MS platform, the 9.2 kDa alpha-1 chain was also detected by Goncalves et al. [9]. Unlike our own observations, they found the 9.2 kDa peak to be overexpressed in sera of high-risk primary breast cancer patients (n = 81) experiencing a relapse versus long-term disease free survivors [9]. The absolute intensities of the m/z 9192 peak in SELDI-TOF MS spectra of Goncalves et al. [9] ranged between 0 and 2, and a clear dichotomous peak intensity distribution was not observed. These discrepancies from our initial findings most likely originate in the serum pre-fractionation that was performed in this study. During protein purification, we repeatedly found the 9.2 kDa peak to be predominantly present in the pH 5 fraction. Goncalves et al. [9] however, subjected only the pH 9/flow through, pH 4, and organic solvent fractions to SELDI-TOF MS analysis, resulting in a suboptimal assay for haptoglobin alpha-1 detection.

Another association between protein expression and recurrence free survival in breast cancer has previously been reported by Kuhajda et al. [22,23]. They described a decreased tumour tissue expression of haptoglobin-related-protein, quantitated immunohistochemically, to be associated with a prolonged recurrence free survival in 70 breast cancer patients [22]. Their findings differ from our observations by the biological matrix analysed (tumour tissue vs. serum), by the exposure used for prediction of recurrence free survival (protein expression vs. phenotype), and by the identity of the protein used for prognostication (haptoglobin-related-protein vs. haptoglobin). Although coded for by two different genes, both proteins have more than 90% amino acid sequence homology. There are, however, distinct differences between the alpha-1 chain of haptoglobin and haptoglobin-related-protein, due to 8 amino acid substitutions [24]. Amino acid sequencing of the m/z 9198 marker, taking 7 out of 8 amino acid substitutions into account, enabled us to unequivocally identify our candidate biomarker as haptoglobin alpha-1 chain (Figure 4B).

The haptoglobin phenotype has not been identified as a predictor of recurrence free survival in breast cancer thus far. The phenotype has nevertheless been associated with clinical outcome of other pathologies, a.o. mortality [25], nephropathy [26], and cardiovascular disease outcome in diabetic patients [27], mortality in HIV infection [28] and mortality in tuberculosis [29]. In these studies, the Hp 2-2 phenotype was invariably associated with worse clinical outcome, in contrast though to the study of Depypere et al. [30], who found the Hp 1-1 phenotype associated with more severe hypertension and proteinuria in patients with preeclampsia.

Despite the potential biological justifications, our promising initial result of the haptoglobin phenotype being a predictor of recurrence free survival in a limited number (n = 63) of high-risk primary breast cancer patients was not confirmed following validation by analysis of a six-fold larger sample set (n = 371). It is unlikely that our findings result from differences in patient characteristics between our discovery and validation sample set, since all (known) characteristics were similarly distributed between both sample sets.

The two major threats to validity of discovery-based proteomics research come from chance and bias [31]. Sources of bias include differences in sample collection and storage, or in analysis [32]. The haptoglobin phenotype however, is not influenced by specimen collection and storage. Besides, the native 1D-gelelectrophoresis method for assessment of haptoglobin phenotype is robust and reproducible [33]. Study results are therefore unlikely to have been influenced by bias, but rather result from chance.

Due to small sample sizes and the artifice of discovery strategies, many biomarker candidates are prone to be false positive, *i.e*. be a type I error (erroneous rejection of the null hypothesis). The chance of candidate biomarkers being type I errors is inferred by the fact that most proteomic datasets are subject to both the 'curse of dimensionality' (large number of features) and the 'curse of dataset sparsity' (limited number of samples) [34]. As such, datasets are frequently subjected to multiple testing in search for candidate biomarkers. Yet, even a level of significance for type I errors of 0.01 is no guarantee that false positive findings are debarred, even following correction for multiple testing. Problems caused by chance are best avoided by analysis of an independent validation dataset, in which false positive markers will be ruled out, as they are unique to the discovery sample set [32,35].

The above-mentioned hurdles in proteomics research apply equally to all other '-omics' research (*e.g*. genomics and metabolomics), as in general, these research approaches suffer from a limited number of samples in comparison with the large number of generated features.

## Conclusion

In conclusion, although we initially found the haptoglobin phenotype to be a predictor of recurrence free survival in a limited number of high-risk primary breast cancer patients, this was not confirmed following validation by analysis of a similar, but six-fold larger sample set. Clearly, validation of initial results is of pivotal importance in determining the clinical significance of a candidate biomarker. In spite of this, few, if any, related clinical diagnostic tests have yet been validated for clinical use, although the number of papers reporting on candidate protein biomarkers is large and still expanding. This lack of validation can result in chance results and erroneous conclusions, leading to disappointment when results cannot be reproduced [36]. Distillation of true positives from the total pool of candidate biomarkers is the single greatest challenge in biomarker development, and should therefore be the emphasis in data-driven proteomics research.

## Competing interests

The authors declare that they have no competing interests.

## Authors' contributions

MCWG participated in the design of the study, performed the laboratory work, and drafted the manuscript. MCWG, HvT, and JWRT performed statistical analyses. NH performed the protein identification. MB, RQGCMH, MAN, SR, PNS, VCGTH, and EGEdV made substantial contributions to acquisition of the samples and the clinical data. SR, JHMS, and JHB conceived of the study. JHMS and JHB participated in the design of the study, and helped to draft the manuscript. All authors were involved in revising the manuscript critically, and all authors read and approved the final manuscript.

## Pre-publication history

The pre-publication history for this paper can be accessed here:


